# Effects of sodium-glucose cotransporter-2 inhibitors on chronic kidney disease progression: a multi-state survival model

**DOI:** 10.1186/s13098-024-01522-6

**Published:** 2024-11-23

**Authors:** Amarit Tansawet, Panu Looareesuwan, Htun Teza, Sarinya Boongird, Gareth J. McKay, John Attia, Oraluck Pattanaprateep, Ammarin Thakkinstian

**Affiliations:** 1 Department of Research and Medical Innovation, Faculty of Medicine Vajira Hospital, Navamindradhiraj University, Bangkok, Thailand; 2 Department of Clinical Epidemiology and Biostatistics, Faculty of Medicine Ramathibodi Hospital, Mahidol University, 4th Floor Sukho Place Building, 218/11 Sukhothai Road, Dusit, Bangkok, 10300 Thailand; 3 Department of Medicine, Faculty of Medicine Ramathibodi Hospital, Mahidol University, Bangkok, Thailand; 4 Centre for Public Health, School of Medicine, Dentistry and Biomedical Sciences, Queen’s University Belfast, Belfast, UK; 5 School of Medicine and Public Health, and Hunter Medical Research Institute, University of Newcastle, New Lambton, NSW Australia

**Keywords:** Sodium-glucose cotransporter-2 inhibitors, Diabetes, Chronic kidney disease

## Abstract

**Background:**

Current guidelines recommend good glycemic control in patients with type 2 diabetes (T2D) to limit the progression of associated complications with combination therapies. This study aimed to compare the rate of chronic kidney disease (CKD) progression between patients who did or did not receive sodium-glucose cotransporter-2 inhibitors (SGLT2i) using a multistate model with two intermediate states (i.e., CKD stage 4 (CKD4) and 5 (CKD5)) and one absorbing state (i.e., death).

**Methods:**

Data from patients with T2D and CKD stage 3 (CKD3) were retrieved for analysis. Patients treated with SGLT2i were matched 1:2 by prescription date with non-SGLT2i patients. The multistate model was constructed from Cox survival regression models specific to each transition stage. Cumulative failure and transition probabilities were estimated from bootstrapping.

**Results:**

Data from 6582 patients (2194 and 4388 patients in the SGLT2i and non-SGLT2i groups, respectively) were analyzed. At 10-year follow-up, patients in the SGLT2i group were more likely to remain at CKD3 compared to the non-SGLT2i group: 82.3% (95% CI 79.9%, 84.6%) vs 60.4% (57.6%, 63.4%). Transition probabilities to CKD4, CKD5, and death were lower in the SGLT2i group than non-SGLT2i group: 11.3% (9.5%, 13.3%) vs 19.8% (17.4%, 22.2%), 2.4% (1.5%, 3.4%) vs 7.4% (5.8%, 9.0%), and 4.1% (2.9%, 5.3%) vs 12.4% (10.3%, 14.6%), respectively.

**Conclusion:**

SGLT2i may delay the decline in renal function and slow CKD progression compared to standard care without SGLT2i.

**Supplementary Information:**

The online version contains supplementary material available at 10.1186/s13098-024-01522-6.

## Introduction

Type 2 diabetes (T2D) is a common noncommunicable disease that places increasing burden on healthcare systems, patients and their families globally [1]. Inadequate blood glucose control commonly leads to severe complications including microvascular (such as chronic kidney disease (CKD) and diabetic retinopathy (DR)) and macrovascular diseases (such as cardiovascular disease (CVD)). T2D is recognized as the main risk factor for CKD, with reported increases of 74% in T2D-related CKD between 1990 and 2017 [2]. International clinical practice guidelines recommend metformin as a first-line treatment, with additional treatment options if insufficient blood glucose control is achieved or if patients are at increased risk of developing T2D-related vascular complications [3].

Meta-analyses have provided evidence of the efficacy of sodium-glucose cotransporter-2 inhibitor (SGLT2i) [4–9], a new class of second-line medication for glycemic control, to reduce the rate of CKD progression in patients with T2D; real-world data showed a reduction in CKD risk of 9.5–14.2% relative to other second-line medications [10]. Recently, the beneficial effects of SGLT2i have also been reported in nondiabetic patients through reduced glomerular hypertension independent of glycemic control [11, 12].

Progression of CKD can be analyzed using multistate models. Patients diagnosed with early-stage CKD (i.e., CKD stage 3 (CKD3)) may progress to more advanced CKD stage 4 (CKD4), 5 (CKD5), and premature death. Although the reno-protective effects of SGLT2i have been reported in patients with T2D, the rate of progression through the various CKD stages [13] mediated by SGLT2i in real world data is not well described. Therefore, the aim of this cohort study was to investigate the effects of SGLT2i on multistate CKD progression using real-world data from Thai patients with T2D and CKD3.

## Methods

**Fig. 1:**
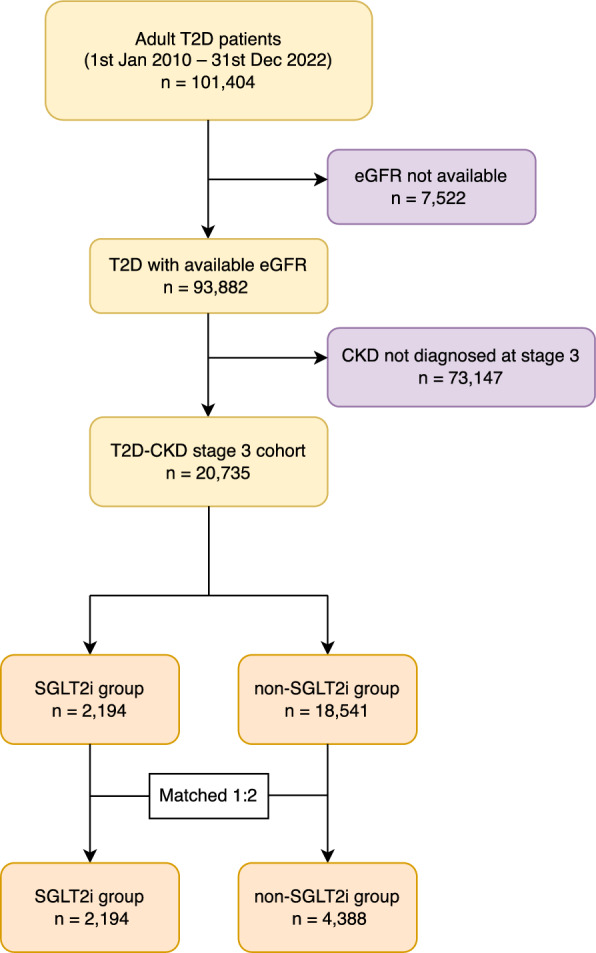
Flow of patient inclusion

This study included a retrospective cohort of patients diagnosed with T2D in Ramathibodi Hospital from 1st January 2010 to 2019 with follow up available until 31st December 2022 (see Fig. 1). T2D was identified from hospital records according to the International Statistical Classification of Diseases, 10th revision (ICD-10), consecutive fasting blood glucose ≥ 126 mg/dl, or glycated hemoglobin (HbA1C) ≥ 6.5%, or commonly prescribed T2D medications as per our previous publication [10]. Adult T2D patients were eligible for inclusion if they were diagnosed with CKD (identified by ICD-10) or if they had an estimated glomerular filtration rate (eGFR) < 60 ml/min/1.73 m^2^ (estimated by the 2021 CKD-EPI equation [14]) recorded persistently for 3 months or longer, see Fig. 1. Patients were excluded if they were previously diagnosed with CKD4 (eGFR 15–29 ml/min/1.73 m^2^), CKD5 (eGFR < 15 ml/min/1.73 m^2^) or received any renal replacement therapy at the time of T2D diagnosis, or had no available eGFR data. Patients receiving SGLT2i were matched 1:2 with patients receiving other second-line antihyperglycemic medications using the earliest prescription date within a 3-year time window.

Clinical, medical, and laboratory databases were linked using encrypted patient hospital numbers to identify the T2D-CKD cohort. This study was approved by the Institutional Review Board of the Faculty of Medicine Ramathibodi Hospital, Mahidol University (MURA2020/125). No informed consent was required regarding retrospective data collection.

### Treatments, outcomes, and covariates

We compared any of SGLT2is (i.e., Dapagliflozin, Canagliflozin, Empagliflozin, and Luseogliflozin) with second-line antihyperglycemic medications (i.e., sulfonylureas, thiazolidinediones, or dipeptidyl peptidase-4 inhibitors), prescribed before progression of CKD3, according to T2D treatment guidelines. Outcomes of interests included CKD3 progression to stages CKD4, CKD5, and/or death. In-hospital death was identified from hospital databases. Some baseline covariate data were missing, ranging from 19.6% (height) to 41.1% (high-density lipoprotein cholesterol (HDL-C) level), see Additional file 1: Table S1. Multiple imputation of missing data by chained equations were performed with 70 replications, assuming data were missing at random.

### Statistical analysis

**Fig. 2:**
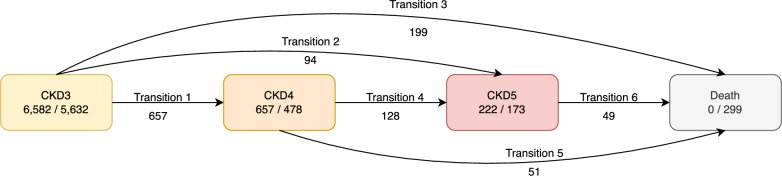
Multistate model of chronic kidney disease progression. The numbers in each box represent the number of patients within and remaining in each state; the numbers under each transition state represent the number of patients moving between states

Baseline characteristics were described by mean and standard deviation (SD) or median and interquartile range (IQR) for continuous variables, and number and percentage for categorical variables. Development of a multistate model was based on four disease states, i.e., the initial state (CKD3), two intermediate states (CKD4 and CKD5), and the absorbing state (death) (Fig. 2). One directional transition was assumed for each state resulting in a total of six transitions: CKD3→CKD4, CKD3→CKD5, CKD3→death, CKD4→CKD5, CKD4→death, and CKD5→death. Failure functions for each transition were subsequently estimated from a semiparametric Cox survival model.

This study was analyzed using an intention-to-treat (ITT) approach. Patients initiating SGLT2i before CKD progression were counted in the SGLT2i group regardless of how long they received this medication; otherwise, patients were included in the non-SGLT2i group. Propensity score (PS) analysis was applied to estimate the treatment effect of SGLT2i on CKD progression as follows: First, a logit treatment model was constructed to estimate PS by regression of the SGLT2i variable on covariates (i.e., age, sex, body mass index (BMI), hemoglobin A1c (HbA1c) level, HDL-C level, baseline eGFR, hypertension, CVD, DR, and health insurance scheme access). Covariate balance was checked to ensure the weighted standardized mean differences did not exceed 0.2 [15]. Second, a semiparametric Cox model was constructed by fitting the SGLT2i variable against time to CKD progression weighted by PS and 1-PS for SGLT2i and non-SGLT2i groups, respectively. Modeling with time interaction was applied in a transition if the proportional hazards assumption was violated. Hazard ratios (HR) along with 95% confidence interval (CI) were estimated. Transition probabilities were estimated for each transition state using a cumulative failure function with 1000-replication bootstrapping. All multistate predictions were performed under *mstatecox* and *multistate* packages [16, 17] using STATA version 18 (StataCorp, Texas, USA). A *P*-value < 0.05 was considered statistically significant.

## Results

### Baseline characteristics

A total of 6582 patients with CKD and T2D were included in this analysis (2194 patients in the SGLT2i group and 4388 patients in the non-SGLT2i group) with median follow-up time (IQR) of 44.9 (18.3, 82.9) months, see Fig. 1. Patient summary characteristics are described in Table 1. Mean age (SD) was 68.6 (10.4) years and 53% of patients were male. Most patients were overweight with median BMI (IQR) of 26.5 (24.3, 28.9) kg/m^2^, and a median HbA1c (IQR) of 7.0% (6.5%, 8.0%). Approximately 33% of patients had abnormal HDL-C levels. Hypertension was common (92%) and frequency of CVD and DR were 26 and 9.4%, respectively.

**Table 1:** Baseline characteristics of patients with type 2 diabetes and chronic kidney disease stage 3

Baseline Characteristics	Overall (N = 6582)	SGLT2i (n = 2194)	Non-SGLT2i (n = 4388)	*p*-value
Age, year, mean (SD)	68.6 (10.4)	66.8 (9.5)	69.5 (10.8)	< 0.001
Age, year, n (%)				
< 40	62 (0.9)	14 (0.6)	48 (1.1)	< 0.001
40–60	1296 (19.7)	519 (23.7)	777 (17.7)	
> 60	5224 (79.4)	1661 (75.7)	3563 (81.2)	
Sex, male, n (%)	3503 (53.2)	1253 (57.1)	2250 (51.3)	< 0.001
BMI, kg/m^2^, median (IQR)	26.5 (24.3, 28.9)	27.3 (25.0, 29.7)	26.1 (23.9, 28.5)	< 0.001
BMI, kg/m^2^, n (%)				
< 18	49 (0.7)	14 (0.6)	35 (0.8)	< 0.001
18–25	2121 (32.2)	530 (24.2)	1591 (36.3)	
> 25	4412 (67.0)	1650 (75.2)	2762 (62.9)	
eGFR, ml/min/1.73 m^2^, mean (SD)	48.9 (8.1)	50.4 (7.4)	48.2 (8.3)	< 0.001
HbA1c, %, median (IQR)	7.0 (6.5, 8.0)	7.3 (6.6, 8.3)	6.9 (6.4, 7.8)	< 0.001
HbA1c, %, n (%)				
< 7	3231 (49.1)	871 (39.7)	2360 (53.8)	< 0.001
≥ 7	3351 (50.9)	1323 (60.3)	2028 (46.2)	
HDL-C, mg/dL, median (IQR)	43.1 (38.0, 50.1)	42.0 (37.0, 49.0)	44.0 (38.0, 51.0)	< 0.001
HDL-C, mg/dL, n (%)				
< 40	2187 (33.2)	830 (37.8)	1357 (30.9)	< 0.001
≥ 40	4395 (66.8)	1364 (62.2)	3031 (69.1)	
Hypertension, n (%)	6023 (91.5)	2052 (93.5)	3971 (90.5)	< 0.001
CVD, n (%)	1732 (26.3)	836 (38.1)	896 (20.4)	< 0.001
DR, n (%)	621 (9.4)	283 (12.9)	338 (7.7)	< 0.001
Health insurance scheme, n (%)				
Universal coverage	1000 (15.2)	177 (8.1)	823 (18.8)	< 0.001
Social security insurance	180 (2.8)	57 (2.6)	127 (2.9)	
Government officer benefits	4042 (61.4)	1577 (71.9)	2465 (56.2)	
Self-pay/Private insurance	1356 (20.6)	383 (17.5)	973 (22.2)	

BMI, body mass index; CVD, cardiovascular disease; DR, diabetic retinopathy; eGFR, estimated glomerular filtration rate; HbA1c, hemoglobin A1c; HDL-C, high-density lipoprotein cholesterol; IQR, interquartile range; SD, standard deviation

Baseline characteristics differed significantly between SGLT2i and non-SGLT2i groups (see Table 1); patients in the SGLT2i group tended to have poorer prognostic factors compared to those in the non-SGLT2i group, except for eGFR. However, the covariate imbalance was improved following adjustment for the inverse probability, see Additional file 1: Table S2. Of CKD5 patients, dialysis was higher in the SGLT2i group when compared to the non-SGLT2i group (76.3 vs 56.0%: *P*-value = 0.011). Only a single patient in each group received kidney transplantation. In the SGLT2i group, the median (IQR) duration of SGLT2i prescription was 18.0 (7.2, 36.5) months; only 23.5% of SGLT2i patients continued SGLT2i to the end of follow-up (i.e., were fully adherent to therapy in the ITT model).

### CKD progression

Of the 6582 patients included at baseline for the initial state, 657 (10%) progressed to CKD4, 94 (1.4%) to CKD5, and 199 (3.0%) died, leaving the remaining 5632 (85.6%) in the initial CKD3 state at study end (Fig. 2). When patients progressed to the intermediate states (i.e., CKD4 and CKD5), they were at further risk of transition either to CKD5 or death. Of the 657 patients who progressed to CKD4, 128 (19.5%) and 51 (7.8%) further progressed to CKD5 and death, respectively. A total of 49 patients (22.1%) of the 222 at CKD5 died. In total, 299 patients (4.5%) transitioned to the absorbing state (i.e., death) by study end. Among 222 patients who reached CKD5, 132 (59.5%) patients received dialysis, whereas 2 (0.9%) and 88 (39.6%) patients received kidney transplantation and supportive care, respectively.

**Fig. 3:**
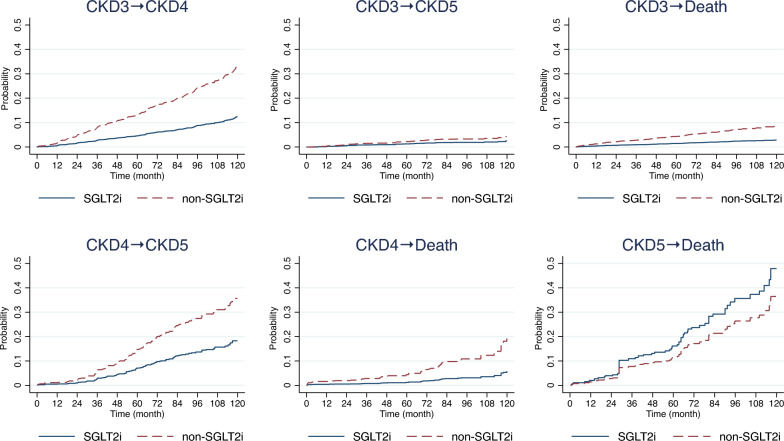
Cumulative failure probability for each transition between states

The cumulative failure and transition probability from the initial CKD3 state to each of the intermediate states and death are described in Additional file 1: Table S3 and S4. Five-year predicted transition probabilities (95% CI) from the initial CKD3 state to CKD4, CKD5, and death were 10.3% (9.4%, 11.3%), 1.8% (1.4%, 2.2%), and 3.4% (2.9%, 4.0%), respectively. For patients that transitioned to CKD4, five-year probabilities for moving to states CKD5 and death were 12.8% (10.2%, 15.9%) and 3.7% (2.4%, 5.6%), respectively. The five-year probability of transitioning from CKD5→death was 12.6% (8.6%, 18.3%). Transition probabilities for CKD progression were also estimated according to whether patients were treated with SGLT2i or not. Treatment with SGLT2i was associated with significantly lower probabilities of progression compared to those in the non-SGLT2i group for all transitions, with the exception of CKD5→death, where the probability was greater for those in the SGLT2i group compared to the non-SGLT2i group, see Fig. 3 and Additional file 1: Table S3. Ten-year probabilities for transition from the baseline CKD3 state to CKD4 and CKD5 were lower in the SGLT2i group compared to the non-SGLT2i group, i.e., 11.3% (9.5%, 13.3%) vs 19.8% (17.4%, 22.2%) and 2.4% (1.5%, 3.4%) vs 7.4% (5.8%, 9.0%), respectively. In addition, patients in the SGLT2i group were less likely to die compared to those in the non-SGLT2i, i.e., 4.1% (2.9%, 5.3%) vs 12.4% (10.3%, 14.6%), see Additional file 1: Table S4.

For patients that received SGLT2i a significant reduction in risk of CKD progression was identified in almost all transitions compared to those that did not receive SGLT2i with HRs (95% CI) of 0.33 (0.26, 0.41; *P*-value < 0.001), 0.57 (0.30, 1.09; *P*-value = 0.087), 0.32 (0.21, 0.50; *P*-value < 0.001), 0.46 (0.25, 0.83; *P*-value = 0.010), and 0.27 (0.11, 0.70; *P*-value = 0.007) for each of the transition states of CKD3→CKD4, CKD3→CKD5, CKD3→death, CKD4→CKD5, and CKD4→death, respectively. Conversely, the risk of transitioning from CKD5→death was 1.44 (0.55, 3.76; *P*-value = 0.459) times greater for those patients in the SGLT2i group compared to the non-SGLT2i group, but this was not significant. For patients undergoing dialysis and supportive care, the SGLT2i group had higher risk of death compared with the non-SGLT2i group with HRs (95% CI) of 1.31 (0.36, 4.83; *P*-value = 0.680) and 1.72 (0.52, 5.70; *P*-value = 0.372) respectively, although this was again not significant.

## Discussion

We conducted a multi-stage analysis to assess the treatment effects of SGLT2i on CKD progression. Our findings highlight the benefits of SGLT2i treatment in patients with T2D and CKD by lowering the probabilities for transition from CKD3 to CKD4, CKD5, and/or death when compared to patients who had not received SGLT2i treatment.

Our findings support those from previous meta-analyses [4–9] that demonstrated the efficacy of SGLT2i in providing renal protection, with a pooled risk reduction between 32 and 45%. Of note, with the exception of the CREDENCE trial [18], the randomized controlled trials that reported renal outcomes (CANVAS program [19], EMPA-REG OUTCOME [20], and DECLARE-TIMI 58 trial [21]), targeted cardiovascular primary endpoints. Therefore, only a minority of patients included in these trials had documented CKD (i.e., eGFR < 60 ml/min/1.73 m^2^) at baseline. Real-world evidence that compared SGLT2i with other second-line medications also highlighted the reno-protective effects of SGLT2i for CKD prevention [10, 22–25]. We previously reported a significant 14.2% reduction in CKD incidence in patients that received SGLT2i compared to those that received sulfonylureas [10]. The reno-protective effects provided by SGLT2i have been observed regardless of eGFR [24, 25]. Our study was novel in the exclusive selection of T2D patients with impaired kidney function at baseline to investigate the efficacy of SGLT2i in reducing the rate of CKD progression in this real-world cohort.

It is also worth noting the speed with which these beneficial effects are noted. Although our models were simulated over a 10-year period, statistically significant effects are seen within the first year for most transitions except CKD5 to death, which lacked statistical significance given the small number of patients included. The speed of the beneficial effects associated with SGLT2i is encouraging for patients and highlights the importance of identifying those who might benefit most from early intervention.

The mode of action of SGLT2i in lowering serum glucose levels is by promoting glucosuria but its reno-protective effect may be mediated through other mechanisms [11, 12]. Natriuresis results following SGLT2i treatment, causing intravascular volume contraction and subsequently reducing blood pressure. By increasing distal tubule sodium delivery, tubuloglomerular feedback is inhibited leading to afferent arteriolar vasoconstriction. These effects lead to a reduction in intraglomerular pressure. In addition, expression of inflammatory biomarkers (such as IL-6, TNF receptor 1, and matrix metalloproteinase 7) are decreased following SGLT2i treatment [26]. Recently, the DAPA-CKD trial [27] showed that SGLT2i could significantly reduce a composite renal outcome [HR (95% CI) = 0.61 (0.51, 0.72)] compared with placebo, independent of glycemic control.

To the best of our knowledge, we are unaware of other studies that have investigated the multistate transitions of CKD-progression following treatment with SGLT2i. Despite the novelty of our study, there were several limitations. The percentage of patients with T2D and CKD receiving SGLT2i in Thailand compared to standard care is still relatively small since its approval in 2015. Moreover, SGLT2i have still to be included within the universal coverage and social security insurance schemes, which support healthcare provision for the majority of the Thai population. As such, the estimate of CKD progression from CKD5 to death is less precise, given the smaller number of affected patients. Although we used propensity score adjustment to balance the effects of covariates between groups, some unknown factors or known factors with unavailable data may exist that could not be controlled for. For example, urinary albumin to creatinine ratio is not routinely measured in our clinical practice and the high rate of missing data could not be sufficiently accounted for in our propensity score computation. Nevertheless, our results are likely to be robust given the bias in our cohort is towards the null, i.e. those receiving SGLT2i had more recognized CKD risk factors compared to those in the control group and a relatively short duration of SGLT2i prescription. Furthermore, given that we could only identify in-hospital mortality, we could have missed those who died in the community. Further large-scale real-world cohort analyses of SGLT2i effects at a population level would prove beneficial in confirming the magnitude of these effects. In addition, personalized prediction models for SGLT2i treatments may provide additional benefits to patients and guide physicians in clinical decision making and best utilization of available resources.

## Conclusions

Provision of SGLT2i may be a more effective treatment option for delaying CKD progression in patients with T2D than other anti-hyperglycemic agents. Longer term evaluation of T2D patients with CKD in receipt of SGLT2i, especially in relation to a cost-effectiveness analysis, will prove beneficial for the evaluation of health outcomes and patient management in resource limited settings.

## Supplementary Information

Additional file 1: Table S1. Percentage of missing covariates. Table S2. Covariate balance before and after inverse probability weight adjustment. Table S3. Percentage cumulative failure probability for each transition across one to ten years of follow-up. Table S4. Percentage transition probabilities for each state across one to ten years of follow-up.

## Data Availability

Data requests should be submitted to the corresponding author (Panu Looareesuwan: panu.loo@mahidol.edu), Prof.Ammarin Thakkinstian (ammarin.tha@mahidol.edu), or at www.CEB-Rama.org. Details of cohort data can also be viewed at this CEB website. A brief proposal will be required along with valuable research question/s and appropriate statistical analysis, which will be evaluated case by case by our research consortiums (consisting of clinical specialists, Clinical Epidemiologists, Biostatisticians, and Data Scientists), the Department of Clinical Epidemiology and Biostatistics, Faculty of Medicine Ramathibodi Hospital, Mahidol University. Once approved, a full proposal is needed to develop and apply for approval from the ethics committee of your local and at the Faculty of Medicine Ramathibodi Hospital, Mahidol University. Further data analysis will be coordinated and/or performed by the local and central data analysts.

## Abbreviations

CI: Confidence interval
CKD: Chronic kidney disease
CVD: Cardiovascular disease
DR: Diabetic retinopathy
eGFR: Estimated glomerular filtration rate
HbA1c: Hemoglobin A1c
HDL-C: High-density lipoprotein cholesterol
HR: Hazard ratio
ICD: International Statistical Classification of Diseases
IQR: Interquartile range
ITT: Intention-to-treat
PS: Propensity score
SD: Standard deviation
SGLT2i: Sodium-glucose cotransporter-2 inhibitor
T2D: Type 2 diabetes

## References

[1] Safiri S, Karamzad N, Kaufman JS, Bell AW, Nejadghaderi SA, Sullman MJM, et al. Prevalence, deaths and disability-adjusted-life-years (DALYs) due to type 2 diabetes and its attributable risk factors in 204 countries and territories, 1990–2019: Results from the Global Burden of Disease study 2019. Front Endocrinol (Lausanne). 2022;13: 838027. 10.3389/fendo.2022.838027. 35282442 10.3389/fendo.2022.838027 PMC8915203

[2] Li H, Lu W, Wang A, Jiang H, Lyu J. Changing epidemiology of chronic kidney disease as a result of type 2 diabetes mellitus from 1990 to 2017: Estimates from Global Burden of Disease 2017. J Diabetes Investig. 2021;12(3):346–56. 10.1111/jdi.13355. 32654341 10.1111/jdi.13355 PMC7926234

[3] ElSayed NA, Aleppo G, Aroda VR, Bannuru RR, Brown FM, Bruemmer D, et al. Summary of revisions: Standards of care in diabetes 2023. Diabetes Care. 2023;46(Suppl 1):S5-9. 10.2337/dc23-Srev. 36507641 10.2337/dc23-Srev PMC9810459

[4] Neuen BL, Young T, Heerspink HJL, Neal B, Perkovic V, Billot L, et al. SGLT2 inhibitors for the prevention of kidney failure in patients with type 2 diabetes: A systematic review and meta-analysis. Lancet Diabetes Endocrinol. 2019;7(11):845–54. 10.1016/S2213-8587(19)30256-6. 31495651 10.1016/S2213-8587(19)30256-6

[5] Zelniker TA, Wiviott SD, Raz I, Im K, Goodrich EL, Bonaca MP, et al. SGLT2 inhibitors for primary and secondary prevention of cardiovascular and renal outcomes in type 2 diabetes: A systematic review and meta-analysis of cardiovascular outcome trials. Lancet. 2019;393(10166):31–9. 10.1016/S0140-6736(18)32590-X. 30424892 10.1016/S0140-6736(18)32590-X

[6] Zelniker TA, Wiviott SD, Raz I, Im K, Goodrich EL, Furtado RHM, et al. Comparison of the effects of glucagon-like peptide receptor agonists and sodium-glucose cotransporter 2 inhibitors for prevention of major adverse cardiovascular and renal outcomes in type 2 diabetes mellitus. Circulation. 2019;139(17):2022–31. 10.1161/CIRCULATIONAHA.118.038868. 30786725 10.1161/CIRCULATIONAHA.118.038868

[7] Lin DS, Lee JK, Hung CS, Chen WJ. The efficacy and safety of novel classes of glucose-lowering drugs for cardiovascular outcomes: A network meta-analysis of randomised clinical trials. Diabetologia. 2021;64(12):2676–86. 10.1007/s00125-021-05529-w. 34536085 10.1007/s00125-021-05529-w

[8] McGuire DK, Shih WJ, Cosentino F, Charbonnel B, Cherney DZI, Dagogo-Jack S, et al. Association of SGLT2 Inhibitors with cardiovascular and kidney outcomes in patients with type 2 diabetes: A meta-analysis. JAMA Cardiol. 2021;6(2):148–58. 10.1001/jamacardio.2020.4511. 33031522 10.1001/jamacardio.2020.4511 PMC7542529

[9] Yamada T, Wakabayashi M, Bhalla A, Chopra N, Miyashita H, Mikami T, et al. Cardiovascular and renal outcomes with SGLT-2 inhibitors versus GLP-1 receptor agonists in patients with type 2 diabetes mellitus and chronic kidney disease: A systematic review and network meta-analysis. Cardiovasc Diabetol. 2021;20(1):14. 10.1186/s12933-020-01197-z. 33413348 10.1186/s12933-020-01197-z PMC7792332

[10] Siriyotha S, Lukkunaprasit T, Looareesuwan P, Nimitphong H, McKay GJ, Attia J, et al. Effects of second-line antihyperglycemic drugs on the risk of chronic kidney disease: Applying a target trial approach to a hospital-based cohort of Thai patients with type 2 diabetes. Cardiovasc Diabetol. 2022;21(1):248. 10.1186/s12933-022-01641-2. 36397062 10.1186/s12933-022-01641-2 PMC9670521

[11] Heerspink HJL, Perkins BA, Fitchett DH, Husain M, Cherney DZI. Sodium glucose cotransporter 2 inhibitors in the treatment of diabetes mellitus: Cardiovascular and kidney effects, potential mechanisms, and clinical applications. Circulation. 2016;134(10):752–72. 10.1161/CIRCULATIONAHA.116.021887. 27470878 10.1161/CIRCULATIONAHA.116.021887

[12] Yau K, Dharia A, Alrowiyti I, Cherney DZI. Prescribing SGLT2 inhibitors in patients with CKD: Expanding indications and practical considerations. Kidney Int Rep. 2022;7(7):1463–76. 10.1016/j.ekir.2022.04.094. 35812300 10.1016/j.ekir.2022.04.094 PMC9263228

[13] Kidney Disease: Improving Global Outcomes (KDIGO) CKD Work Group. KDIGO 2012 Clinical Practice Guideline for the Evaluation and Management of Chronic Kidney Disease Chapter 2: Definition, identification, and prediction of CKD progression. Kidney Int Suppl. 2013;3(1):63–72. 10.1038/kisup.2012.65 10.1038/kisup.2012.65 PMC4089637 25018976

[14] Inker LA, Eneanya ND, Coresh J, Tighiouart H, Wang D, Sang Y, et al. New creatinine- and cystatin C–based equations to estimate GFR without race. N Engl J Med. 2021;385(19):1737–49. 10.1056/NEJMoa2102953. 34554658 10.1056/NEJMoa2102953 PMC8822996

[15] Austin PC. Balance diagnostics for comparing the distribution of baseline covariates between treatment groups in propensity-score matched samples. Stat Med. 2009;28(25):3083–107. 10.1002/sim.3697. 19757444 10.1002/sim.3697 PMC3472075

[16] Metzger SK, Jones BT. mstatecox: A package for simulating transition probabilities from semiparametric multistate survival models. Stata J. 2018;18(3):533–63. 10.1177/1536867X1801800304.

[17] Crowther MJ, Lambert PC. Parametric multi-state survival models: Flexible modelling allowing transition-specific distributions with application to estimating clinically useful measures of effect differences. Stat Med. 2017;36(29):4719–42. 10.1002/sim.7448. 28872690 10.1002/sim.7448

[18] Perkovic V, Jardine MJ, Neal B, Bompoint S, Heerspink HJL, Charytan DM, et al. Canagliflozin and renal outcomes in type 2 diabetes and nephropathy. N Engl J Med. 2019;380(24):2295–306. 10.1056/NEJMoa1811744. 30990260 10.1056/NEJMoa1811744

[19] Neal B, Perkovic V, Mahaffey KW, de Zeeuw D, Fulcher G, Erondu N, et al. Canagliflozin and cardiovascular and renal events in type 2 diabetes. N Engl J Med. 2017;377(7):644–57. 10.1056/NEJMoa1611925. 28605608 10.1056/NEJMoa1611925

[20] Wanner C, Inzucchi SE, Lachin JM, Fitchett D, von Eynatten M, Mattheus M, et al. Empagliflozin and progression of kidney disease in type 2 diabetes. N Engl J Med. 2016;375(4):323–34. 10.1056/NEJMoa1515920. 27299675 10.1056/NEJMoa1515920

[21] Mosenzon O, Wiviott SD, Cahn A, Rozenberg A, Yanuv I, Goodrich EL, et al. Effects of dapagliflozin on development and progression of kidney disease in patients with type 2 diabetes: an analysis from the DECLARE-TIMI 58 randomised trial. Lancet Diabetes Endocrinol. 2019;7(8):606–17. 10.1016/S2213-8587(19)30180-9. 31196815 10.1016/S2213-8587(19)30180-9

[22] Heerspink HJL, Karasik A, Thuresson M, Melzer-Cohen C, Chodick G, Khunti K, et al. Kidney outcomes associated with use of SGLT2 inhibitors in real-world clinical practice (CVD-REAL 3): A multinational observational cohort study. Lancet Diabetes Endocrinol. 2020;8(1):27–35. 10.1016/S2213-8587(19)30384-5. 31862149 10.1016/S2213-8587(19)30384-5

[23] Pasternak B, Wintzell V, Melbye M, Eliasson B, Svensson AM, Franzén S, et al. Use of sodium-glucose co-transporter 2 inhibitors and risk of serious renal events: Scandinavian cohort study. BMJ. 2020;369: m1186. 10.1136/bmj.m1186. 32349963 10.1136/bmj.m1186 PMC7188014

[24] Xie Y, Bowe B, Gibson AK, McGill JB, Maddukuri G, Yan Y, et al. Comparative effectiveness of SGLT2 inhibitors, GLP-1 receptor agonists, DPP-4 inhibitors, and sulfonylureas on risk of kidney outcomes: Emulation of a target trial using health care databases. Diabetes Care. 2020;43(11):2859–69. 10.2337/dc20-1890. 32938746 10.2337/dc20-1890

[25] Lin FJ, Wang CC, Hsu CN, Yang CY, Wang CY, Ou HT. Renoprotective effect of SGLT-2 inhibitors among type 2 diabetes patients with different baseline kidney function: A multi-center study. Cardiovasc Diabetol. 2021;20(1):203. 10.1186/s12933-021-01396-2. 34620182 10.1186/s12933-021-01396-2 PMC8499571

[26] Heerspink HJL, Perco P, Mulder S, Leierer J, Hansen MK, Heinzel A, et al. Canagliflozin reduces inflammation and fibrosis biomarkers: A potential mechanism of action for beneficial effects of SGLT2 inhibitors in diabetic kidney disease. Diabetologia. 2019;62(7):1154–66. 10.1007/s00125-019-4859-4. 31001673 10.1007/s00125-019-4859-4 PMC6560022

[27] Heerspink HJL, Stefánsson BV, Correa-Rotter R, Chertow GM, Greene T, Hou FF, et al. Dapagliflozin in patients with chronic kidney disease. N Engl J Med. 2020;383(15):1436–46. 10.1056/NEJMoa2024816. 32970396 10.1056/NEJMoa2024816

