# Supplementary material for: Effects of sodium-glucose cotransporter-2 inhibitors on chronic kidney disease progression: a multi-state survival model
Source: Diabetol Metab Syndr. 2024 Nov 23;16:281. doi: 10.1186/s13098-024-01522-6 (PMC11585174; doi:10.1186/s13098-024-01522-6)
Supplement: Supplementary file 1 — Additional file 1: Table S1. Percentage of missing covariates. Table S2. Covariate balance before and after inverse probability weight adjustment. Table S3. Percentage cumulative failure probability for each transition across one to ten years of follow-up. Table S4. Percentage transition probabilities for each state across one to ten years of follow-up. [file 13098_2024_1522_MOESM1_ESM.docx]

**Effects of sodium-glucose cotransporter-2 inhibitors on chronic kidney disease progression: A multi-state survival model**

Amarit Tansawet; Panu Looareesuwan; Htun Teza; Sarinya Boongird; Gareth J McKay; John Attia; Oraluck Pattanaprateep; Ammarin Thakkinstian

**Supplementary**

**Table S1.** Percentage of missing covariates page 2

**Table S2.** Covariate balance before and after inverse probability weight adjustment page 3

**Table S3.** Percentage cumulative failure probability for each transition across one to ten years of follow-up page 4

**Table S4.** Percentage transition probabilities for each state across one to ten years of follow-up page 5

**Table S1.** Percentage of missing covariates

| Variable | Missing (%) |
| --- | --- |
| Age | 0.00 |
| Sex | 0.00 |
| Weight | 28.22 |
| Height | 19.59 |
| eGFR | 0.00 |
| HDL-C | 41.13 |
| HbA1c | 28.63 |
| Hypertension | 0.00 |
| CVD | 0.00 |
| DR | 0.00 |

CVD, cardiovascular disease; DR, diabetic retinopathy; eGFR, estimated glomerular filtration rate; HbA1c, hemoglobin A1c; HDL-C, high-density lipoprotein cholesterol

**Table S2.** Covariate balance before and after inverse probability weight adjustment

| Variables | Standardized difference | | Variance ratio | |
| --- | --- | --- | --- | --- |
|  | Raw | Weighted | Raw | Weighted |
| Age, year |  |  |  |  |
| 40 - 60 | 0.147229 | -0.018328 | 1.239632 | 0.9718187 |
| > 60 | -0.1338578 | 0.0166271 | 1.204997 | 0.9756549 |
| Sex (Male) | 0.1172756 | -0.0066177 | 0.9806397 | 1.000767 |
| BMI, kg/m^2^ |  |  |  |  |
| < 18 | -0.0188939 | -0.0037051 | 0.8014692 | 0.9580547 |
| > 25 | 0.2675976 | 0.0068058 | 0.7996457 | 0.99494 |
| eGFR (ml/min/1.73 m^2^) | 0.2801222 | 0.0065615 | 0.7953911 | 0.9300069 |
| HDL-C ≥ 40 mg/dL | -0.1457426 | 0.0001199 | 1.101249 | 0.9999144 |
| HbA1c ≥ 7 % | 0.2850833 | -0.0007748 | 0.96329 | 1.000025 |
| Hypertension | 0.1119595 | -0.0087426 | 0.7040261 | 1.026165 |
| CVD | 0.3961874 | 0.0170563 | 1.45172 | 1.017762 |
| DR | 0.171534 | 0.0003832 | 1.580645 | 1.001074 |
| Health insurance scheme |  |  |  |  |
| Social security insurance | -0.0181262 | -0.0058218 | 0.9005817 | 0.9664094 |
| Government officer benefits | 0.3315896 | -0.0171343 | 0.8212581 | 1.007575 |
| Self-pay/ Private insurance | -0.118535 | 0.0011361 | 0.8351657 | 1.001634 |

BMI, body mass index; CVD, cardiovascular disease; DR, diabetic retinopathy; eGFR, estimated glomerular filtration rate; HbA1c, hemoglobin A1c; HDL-C, high-density lipoprotein cholesterol

**Table S3.** Percentage cumulative failure probability for each transition across one to ten years of follow-up

| Transition | Cohort | Time (years) | | | | | | | | | |
| --- | --- | --- | --- | --- | --- | --- | --- | --- | --- | --- | --- |
|  |  | 1 | 2 | 3 | 4 | 5 | 6 | 7 | 8 | 9 | 10 |
| CKD3🡪CKD4 | Overall | 1.4  (1.1, 1.7) | 3.9  (3.4, 4.5) | 6.5  (5.8, 7.2) | 8.4  (7.6, 9.3) | 10.3  (9.4, 11.3) | 12.9  (11.8, 14.1) | 14.8  (13.6, 16.1) | 18.1  (16.7, 19.7) | 20.0  (18.4, 21.7) | 23.5  (21.5, 25.6) |
|  | SGLT2i | 0.4  (0.1, 0.8) | 1.3  (0.6, 2.0) | 2.2  (1.3, 3.1) | 3.1  (2.2, 4.2) | 4.0  (2.9, 5.3) | 5.6  (4.3, 7.1) | 6.8  (5.3, 8.4) | 8.7  (7.0, 10.5) | 10.2  (8.5, 12.0) | 13.2  (11.2, 15.3) |
|  | Non-SGLT2i | 1.8  (1.0, 2.7) | 5.1  (3.7, 6.5) | 8.5  (6.8, 10.1) | 11.3  (9.5, 13.3) | 13.8  (11.7, 15.9) | 17.7  (15.4, 20.0) | 20.2  (17.8, 22.8) | 23.9  (21.4, 26.5) | 26.5  (23.7, 29.2) | 31.0  (28.3, 33.9) |
| CKD3🡪CKD5 | Overall | 0.2  (0.1, 0.4) | 0.7  (0.5, 1.0) | 1.1  (0.9, 1.5) | 1.2  (0.9, 1.6) | 1.8  (1.4, 2.2) | 2.3  (1.8, 2.9) | 2.5  (2.0, 3.2) | 2.7  (2.1, 3.3) | 2.9  (2.3, 3.7) | 3.2  (2.5, 4.2) |
|  | SGLT2i | 0.2  (0.0, 0.5) | 0.5  (0.1, 1.0) | 0.9  (0.3, 1.5) | 0.9  (0.4, 1.5) | 1.3  (0.7, 2.0) | 1.6  (0.9, 2.5) | 1.8  (1.1, 2.7) | 1.9  (1.1, 2.8) | 2.0  (1.2, 3.0) | 2.5  (1.5, 3.5) |
|  | Non-SGLT2i | 0.3  (0.0, 0.7) | 0.9  (0.4, 1.5) | 1.5  (0.8, 2.3) | 1.6  (0.9, 2.4) | 2.2  (1.3, 3.1) | 2.9  (1.9, 3.9) | 3.2  (2.0, 4.3) | 3.3  (2.2, 4.4) | 3.5  (2.4, 4.7) | 4.2  (3.0, 5.5) |
| CKD3🡪Death | Overall | 1.0  (0.8, 1.3) | 1.6  (1.3, 2.0) | 2.3  (1.9, 2.7) | 2.8  (2.3, 3.3) | 3.4  (2.9, 4.0) | 4.2  (3.6, 4.9) | 4.8  (4.1, 5.6) | 5.4  (4.6, 6.4) | 5.9  (5.0, 6.9) | 6.2  (5.2, 7.3) |
|  | SGLT2i | 0.4  (0.1, 0.8) | 0.7  (0.2, 1.3) | 0.9  (0.4, 1.5) | 1.1  (0.5, 1.9) | 1.4  (0.7, 2.2) | 1.8  (1.0, 2.7) | 2.0  (1.2, 3.0) | 2.5  (1.6, 3.5) | 2.6  (1.7, 3.7) | 2.9  (1.9, 4.0) |
|  | Non-SGLT2i | 1.3  (0.7, 2.1) | 2.1  (1.3, 3.2) | 2.8  (1.8, 3.9) | 3.4  (2.4, 4.7) | 4.3  (3.0, 5.7) | 5.4  (4.0, 6.8) | 6.1  (4.5, 7.8) | 7.3  (5.6, 9.0) | 7.9  (6.1, 9.7) | 8.5  (6.6, 10.3) |
| CKD4🡪CKD5 | Overall | 0.9  (0.4, 2.0) | 2.1  (1.2, 3.5) | 5.7  (4.1, 7.9) | 9.1  (7.0, 11.8) | 12.8  (10.2, 15.9) | 18.2  (15.0, 21.9) | 21.8  (18.2, 25.9) | 24.6  (20.7, 29.1) | 27.5  (23.2, 32.5) | 30.0  (26.0, 36.7) |
|  | SGLT2i | 0.5  (0.1, 1.0) | 1.0  (0.5, 1.7) | 2.7  (1.7, 3.8) | 4.5  (3.2, 5.9) | 7.1  (5.5, 8.7) | 9.8  (7.9, 11.5) | 12.0  (10.1, 14.0) | 13.6  (11.5, 15.6) | 15.6  (13.3, 17.8) | 18.3  (15.9, 20.6) |
|  | Non-SGLT2i | 1.1  (0.5, 1.8) | 2.2  (1.4, 3.1) | 5.9  (4.5, 7.4) | 9.5  (7.8, 11.5) | 14.8  (12.6, 17.0) | 20.1  (17.6, 22.4) | 24.5  (21.7, 27.0) | 27.4  (24.6, 30.1) | 31.0  (28.3, 33.9) | 35.6  (32.8, 38.6) |
| CKD4🡪Death | Overall | 1.4  (0.7, 2.6) | 1.7  (0.9, 3.1) | 2.4  (1.5, 4.0) | 3.2  (2.1, 5.0) | 3.7  (2.4, 5.6) | 5.6  (3.9, 8.1) | 8.7  (6.2, 12.0) | 9.5  (6.9, 13.1) | 10.8  (7.8, 14.8) | 15.2  (10.9, 21.0) |
|  | SGLT2i | 0.5  (0.1, 0.9) | 0.6  (0.1, 1.0) | 0.8  (0.3, 1.4) | 1.0  (0.5, 1.7) | 1.2  (0.6, 1.9) | 1.8  (1.0, 2.7) | 2.8  (1.9, 3.9) | 3.1  (2.1, 4.3) | 3.6  (2.4, 4.8) | 5.7  (4.2, 7.2) |
|  | Non-SGLT2i | 1.6  (0.9, 2.4) | 2.0  (1.2, 2.9) | 2.8  (1.9, 3.8) | 3.7  (2.6, 4.8) | 4.4  (3.2, 5.7) | 6.5  (5.1, 8.0) | 9.8  (8.1, 11.5) | 10.8  (9.1, 12.7) | 12.3  (10.4, 14.2) | 19.3  (16.9, 21.6) |
| CKD5🡪Death | Overall | 2.3  (1.0, 5.4) | 4.2  (2.2, 7.8) | 6.6  (4.0, 10.9) | 8.8  (5.6, 13.7) | 12.6  (8.6, 18.3) | 19.1  (13.9, 26.1) | 23.1  (17.0, 30.9) | 29.1  (21.6, 38.7) | 33.4  (24.6, 44.2) | 40.4  (29.9, 53.0) |
|  | SGLT2i | 2.0  (1.2, 2.9) | 4.0  (2.9, 5.2) | 11.0  (9.1, 12.9) | 13.2  (11.2, 15.2) | 16.1  (14.0, 18.5) | 23.7  (21.2, 26.4) | 29.3  (26.5, 32.0) | 35.7  (32.7, 38.7) | 37.4  (34.4, 40.5) | 48.0  (45.1, 51.2) |
|  | Non-SGLT2i | 1.4  (0.8, 2.1) | 2.8  (1.7, 3.8) | 7.7  (6.2, 9.4) | 9.3  (7.7, 11.0) | 11.5  (9.6, 13.5) | 17.1  (14.8, 19.3) | 21.3  (18.8, 23.6) | 26.3  (23.7, 28.9) | 27.7  (25.0, 30.2) | 36.3  (33.5, 39.1) |

CKD3, chronic kidney disease stage 3; CKD4, chronic kidney disease stage 4; CKD5, chronic kidney disease stage 5; SGLT2i, sodium-glucose co-transporter 2 inhibitor

Overall cohort’s cumulative failure probabilities were approximated from Kaplan-Meier estimators at the exact or closest time. Parenthesis indicates 95% confidence interval.

**Table S4.** Percentage transition probabilities for each state across one to ten years of follow-up

| State | Cohort | Time (years) | | | | | | | | | |
| --- | --- | --- | --- | --- | --- | --- | --- | --- | --- | --- | --- |
|  |  | 1 | 2 | 3 | 4 | 5 | 6 | 7 | 8 | 9 | 10 |
| CKD3 | SGLT2i | 99.0  (98.4, 99.5) | 97.5  (96.5, 98.4) | 96.0  (94.7, 97.2) | 94.9  (93.5, 96.2) | 93.4  (91.9, 94.9) | 91.2  (89.4, 92.9) | 89.6  (87.7, 91.5) | 87.3  (85.3, 89.3) | 85.6  (83.5, 87.7) | 82.3  (79.9, 84.6) |
|  | Non-SGLT2i | 96.7  (95.6, 97.8) | 92.0  (90.3, 93.7) | 87.6  (85.6, 89.7) | 84.3  (82.1, 86.5) | 80.7  (78.2, 83.1) | 75.7  (73.2, 78.3) | 72.6  (70.0, 75.4) | 68.2  (65.4, 71.1) | 65.3  (62.5, 68.4) | 60.4  (57.6, 63.4) |
| CKD4 | SGLT2i | 0.4  (0.1, 0.9) | 1.3  (0.6, 2.0) | 2.2  (1.3, 3.2) | 3.0  (2.1, 4.1) | 3.8  (2.7, 4.9) | 5.2  (3.8, 6.6) | 6.1  (4.6, 7.6) | 7.8  (6.2, 9.4) | 9.0  (7.4, 10.8) | 11.3  (9.5, 13.3) |
|  | Non-SGLT2i | 1.8  (1.0, 2.6) | 5.0  (3.7, 6.5) | 7.9  (6.3, 9.6) | 10.1  (8.5, 12.1) | 11.7  (9.8, 13.7) | 14.2  (12.1, 16.3) | 15.1  (13.0, 17.3) | 17.7  (15.4, 19.9) | 18.7  (16.4, 21.1) | 19.8  (17.4, 22.2) |
| CKD5 | SGLT2i | 0.2  (0.0, 0.4) | 0.5  (0.1, 1.0) | 0.8  (0.3, 1.4) | 0.9  (0.4, 1.6) | 1.3  (0.7, 2.1) | 1.6  (0.9, 2.5) | 1.8  (1.1, 2.7) | 1.8  (1.0, 2.7) | 2.1  (1.3, 3.0) | 2.4  (1.5, 3.4) |
|  | Non-SGLT2i | 0.3  (0.0, 0.6) | 0.9  (0.4, 1.5) | 1.6  (0.9, 2.5) | 2.1  (1.3, 3.0) | 3.1  (2.1, 4.2) | 4.3  (3.1, 5.6) | 5.1  (3.8, 6.5) | 5.5  (4.1, 6.9) | 6.5  (5.0, 8.0) | 7.4  (5.8, 9.0) |
| Death | SGLT2i | 0.4  (0.1, 0.9) | 0.7  (0.3, 1.3) | 1.0  (0.4, 1.6) | 1.2  (0.6, 2.0) | 1.5  (0.8, 2.4) | 2.0  (1.2, 2.9) | 2.4  (1.5, 3.5) | 3.0  (2.0, 4.1) | 3.3  (2.2, 4.5) | 4.1  (2.9, 5.3) |
|  | Non-SGLT2i | 1.3  (0.6, 2.0) | 2.1  (1.2, 3.0) | 2.8  (1.8, 3.9) | 3.5  (2.4, 4.8) | 4.4  (3.2, 5.8) | 5.9  (4.4, 7.3) | 7.2  (5.5, 8.9) | 8.7  (6.9, 10.5) | 9.5  (7.7, 11.4) | 12.4  (10.3, 14.6) |

CKD3, chronic kidney disease stage 3; CKD4, chronic kidney disease stage 4; CKD5, chronic kidney disease stage 5; SGLT2i, sodium-glucose co-transporter 2 inhibitor

Parenthesis indicates 95% confidence interval.
